# Supplementary material for: Mitigating CaCO3 crystal nucleation and growth through continuous ion displacement via alternating electric fields
Source: Nat Commun. 2025 Jan 2;16:35. doi: 10.1038/s41467-024-55176-z (PMC11697440; doi:10.1038/s41467-024-55176-z)
Supplement: Supplementary file 1 — Supplementary Information [file 41467_2024_55176_MOESM1_ESM.pdf]

SUPPLEMENTARY INFORMATION (SI)

Mitigating $CaCO_3$ crystal nucleation and growth through continuous ion displacement via alternating electric fields

Yiming Liu[1,2,3]†, Minhao Xiao[1]†, Xiaochuan Huang[2,3], Jane Park[4], Matthew E. Hoffman[1], Yuren Feng[2,3], Alicia Kyoungjin An[5], Qilin Li[2,3,6,7], Eric M.V. Hoek[1,8,9,10]* & David Jassby[1,8,9]*

[1] Department of Civil & Environmental Engineering, University of California Los Angeles (UCLA), Los Angeles, CA, USA
[2] National Science Foundation (NSF) Nanosystems Engineering Research Center for Nanotechnology-Enabled Water Treatment, Rice University, Houston, TX, USA
[3] Department of Civil & Environmental Engineering, Rice University, Houston, TX, USA
[4] Department of Chemical & Biomolecular Engineering, UCLA, Los Angeles, CA, USA
[5] Department of Chemical & Biological Engineering, The Hong Kong University of Science and Technology, Hong Kong SAR, China
[6] Department of Chemical & Biomolecular Engineering, Rice University, Houston, TX, USA
[7] Department of Materials Science & NanoEngineering, Rice University, Houston, TX, USA
[8] California NanoSystems Institute, UCLA, Los Angeles, CA, USA
[9] Institute of the Environment & Sustainability, UCLA, Los Angeles, CA, USA
[10] Energy Science & Distributed Resources Division, Lawrence Berkeley National Laboratory, Berkeley, CA, USA

Prepared for

Nature Communications

†These authors contributed equally to this work
*Joint corresponding author emails: emvhoek@ucla.edu; jassby@ucla.edu

Summary of Pages, Tables and Figures
18 Pages, 4 Tables, 7 Figures.

Summary of Contents

- Supplementary Note 1. Scope comparison of this study and previous AC scale prevention studies
- Supplementary Note 2. Specific capacitance in capacitive deionization
- Supplementary Note 3. Real-time AC voltage data
- Supplementary Note 4. Summary of $CaCO_3$ scale prevention data
- Supplementary Note 5. Long-term evaluation of $CaCO_3$ homogeneous precipitation prevention
- Supplementary Note 6. Additional surface characterization data
- Supplementary Note 7. Comparison between measured and simulated electric currents
- Supplementary Note 8. Derivation of simplified Nernst-Planck equation
- Supplementary Note 9. Brownian motion calculations
- Supplementary Note 10. Impact of EDL charging on currents
- Supplementary Note 11. Physics-based crystallization model

Supplementary Note 1. Scope comparison of this study and previous AC scale prevention studies

The scope of this study is compared to previously published AC scale prevention studies. The relevant aspects of previous studies are summarized in Table S1. Critically, we explore the impact of AC potential on both homogeneous nucleation and heterogeneous nucleation, while other studies primarily are focused on the heterogeneous nucleation on the surfaces. By demonstrating its impact on homogeneous nucleation, we enable the possibilities to disrupt any crystal nucleation processes through application of alternating electric fields.

Supplementary Table 1. The comparison between this study and other studies on the scope of investigation.

Reference	Nucleation	Electrical conditions	Mechanistic model	Application scenario
This study	Homogeneous nucleation, heterogeneous nucleation	Voltage, frequency	Quantitative model on equivalent circuit, ion displacement, and nucleation	Heat exchangers
Rao et al.[1]	Heterogeneous nucleation	Frequency	Qualitative model	Membranes
Jung et al.[2]	Heterogeneous nucleation	Single condition	Qualitative model	Membranes
Kim et al.[3]	Heterogeneous nucleation	Voltage, frequency, waveform	Quantitative model on ion displacement	Membranes

Supplementary Note 2. Specific capacitance in capacitive deionization

The specific capacitance (C_m, F g^{-1}) is determined by:

$$C_m = \frac{C_d}{m} \tag{1}$$

where C_d (F) is the EDL capacitance and m (g) is the mass of the electrode. The electrode materials and specific electrode capacitance in the present study and CDI studies are shown in Table S2. The specific capacitance values of the CDI electrode (different forms of porous carbon) are consistently 3 orders-of-magnitude higher than that of our titanium electrode,

which leads to the charging time in the scale of hours (while our charging time scale is fractions of a second).

Supplementary Table 2. The comparison between the electrode in this study and the CDI electrodes.

Reference	Electrode materials	Specific capacitance (F g^{-1})	Charging time (s)
This study	Solid titanium plate	0.19	0.12
Dong et al.[4]	Reduced Graphene Oxide/Activated Carbon Nanofibers	256	6000
Laxman et al.[5]	Activated carbon cloth/ZnO nanorod	100	1000
Li et al.[6]	Ordered mesoporous carbons	192	7200

Supplementary Note 3. Real-time AC voltage data

To document the dynamics of EDL charging under different conditions, real-time AC voltage measurements were conducted using an oscilloscope. These measurements captured under different frequency and water chemistry conditions are presented in Fig. S1a-c. When the EDL reached full charge in an AC half-period, the absolute voltage value reached a steady-state maximum corresponding to an applied potential (e.g., achieving 2 V with 4 V_{pp} AC potential) by the end of the half-period. In the cases of higher frequencies ranging from 10 to 100 Hz, the absolute value of voltage kept increasing (i.e., constantly charging) during each half-period, as (Fig. S1a), as the EDL failed to achieve full charge. This phenomenon indicated that at higher frequencies, the EDL exhibits the characteristics of a conductor, maintaining stable currents under 4 V_{pp} at both 10 and 100 Hz potentials. In contrast, at a reduced frequency of 1 Hz, the voltage first increased and then stabilized by the end of a half-period (Fig. S1b), which indicates the capacitive characteristic of EDL at lower frequencies.

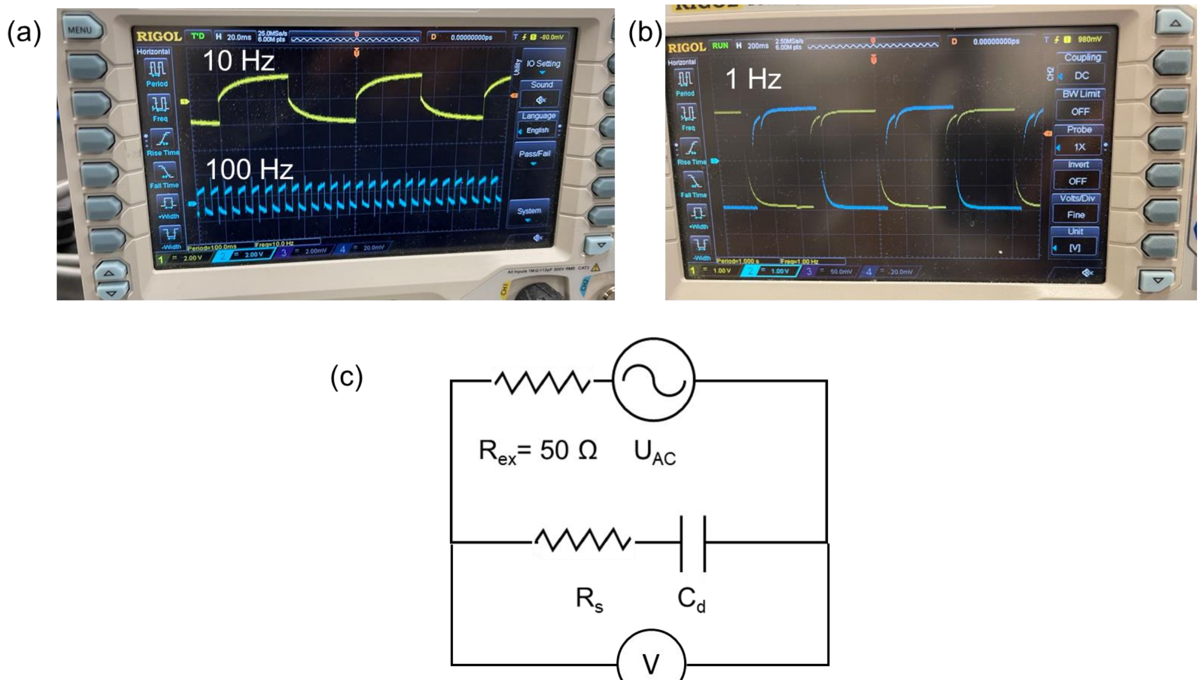

Supplementary Fig. 1. Readings and placement of oscilloscope within the electrochemical system. a,**b**, Voltage as a function of time under the conditions of 4 V_{pp}, 10 Hz (yellow) and 4 V_{pp}, 100 Hz (blue) AC potential (**a**), and 4 V_{pp}, 1 Hz AC potential only (yellow and blue) (**b**). **c**, the placement of oscilloscope as a voltmeter in parallel with the combined solution resistance (R_s) and EDL capacitance (C_d) in the equivalent circuit.

Supplementary Note 4. Summary of $CaCO_3$ scale prevention data

To summarize the effects of AC frequency and voltage, we used relative turbidity as defined in Eq. 20 of manuscript. Fig. S2 presents the relative turbidity against a range of frequencies from 0 to 100 Hz and voltages from 4 to 20 V_{pp}. We observed that increasing the frequency to 0.1 Hz led to a 97% decrease in relative turbidity, and from 10 Hz resulted in 70% increase in relative turbidity, respectively. In contrast, changes in voltage did not significantly impact relative turbidity until reaching 20 V_{pp}, at which point we saw an 82% reduction in relative turbidity. This indicated a significant effect on scale prevention at this higher voltage level.

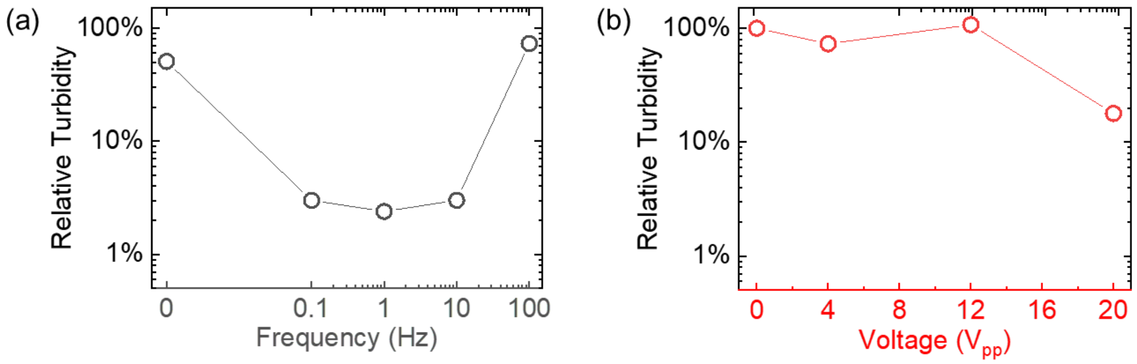

Supplementary Fig. 2. Relative precipitation under different electrical conditions. a,b, Relative turbidity as a function of frequency (a) and voltage (b) of all the electrical conditions.

Supplementary Note 5. Long-term evaluation of $CaCO_3$ homogeneous precipitation prevention

We explored the long-term impact of AC potentials (i.e., 4 V_{pp}, 1 Hz) on $CaCO_3$ bulk precipitation. The bulk turbidity, pH, and conductivity of $CaCO_3$ supersaturated solution were measured over a 24-hour duration. The $CaCO_3$ bulk precipitation was successfully mitigated over the long-term. Specifically, turbidity at 4 V_{pp}, 1 Hz was consistently orders-of-magnitude lower than the control, increasing from 0.17±0.01 to 0.29±0.02 NTU over the 2-to-24-hour period, compared to the control (0 V), where turbidity increased from 8.54±1.12 to 80.70±2.32 NTU (Fig. S3a). Critically, the bulk turbidity at 4 V_{pp}, 1 Hz reached only 0.29±0.02 NTU after 24 hours, indicating excellent performance even with a high supersaturation index of 11.04.

During the 24-hour period, the conductivity (left y-axis) decreased due to both CO_2 diffusion and $CaCO_3$ precipitation, while the decline in pH (right y-axis) was primarily influenced by the CO_2 diffusion (Fig. S3b). Conductivity values during the first four hours of the experiment, decreased from 2518±13 to 2010±56 μS cm^{-1} at 0 V, and from 2508±22 to 2087±43 μS cm^{-1} at 4 V_{pp}, 1 Hz – statistically identical values. This suggested that the decline in conductivity was mainly due to CO_2 diffusion into the water. From 4 to 24 hours, conductivity values at 4 V_{pp}, 1 Hz decreased from 2060±40 to 1497±28 μS cm^{-1} while at 0 V, they dropped from 1951±64 to 1313±58 μS cm^{-1}) (Fig. S3b). The more significant decrease in

conductivity after 4 hours was caused by the $CaCO_3$ precipitation, as evidenced by the dramatic increase in bulk turbidity. In contrast, the pH values were similar between 4 V_{pp}, 1 Hz (from 11.97±0.02 to 10.76±0.06) and 0 V (from 11.95±0.02 to 10.64±0.09) over the 24-hour period, declining in a near-linear fashion (Fig. S3b). The decrease in pH reduced the concentration of CO_3^{-2} for $CaCO_3$ precipitation, mitigating the initial sharp increase in bulk turbidity after 4 hours and leading to a plateau between 4 to 24 hours (Fig. S3a).

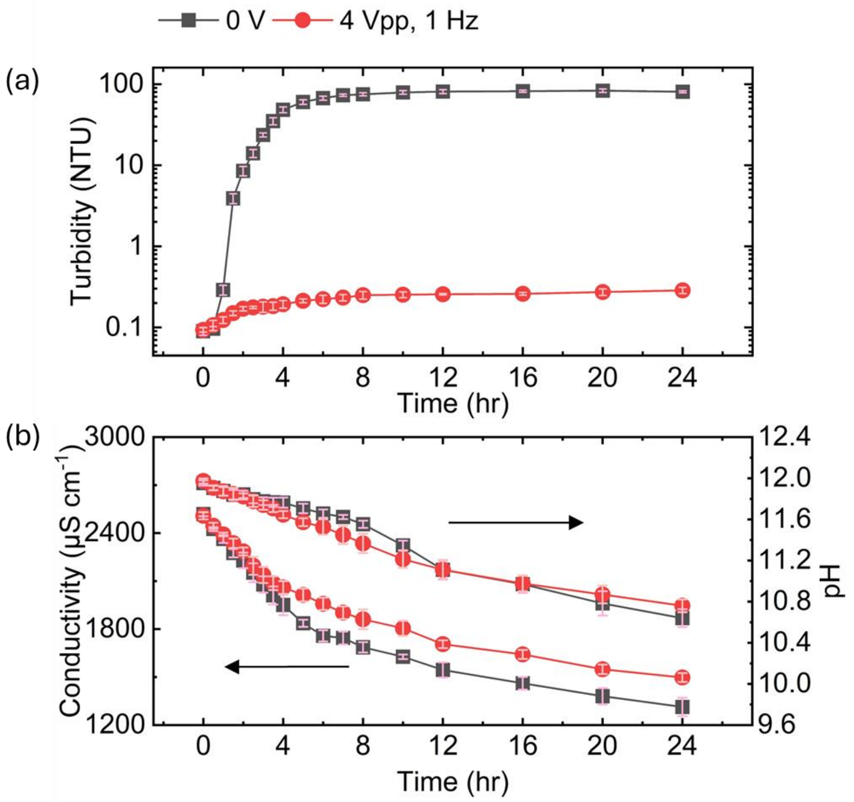

Supplementary Fig. 3. Long-term evolution of precipitation. a,b, Turbidity (**a**), pH and conductivity (**b**) as a function of time from 0 to 24 hours at 0 V and 4 V_{pp}, 1 Hz AC potential.

Supplementary Note 6. Additional surface characterization data

To investigate the $CaCO_3$ coverage at a microscopic level, we compared SEM micrographs under the conditions of 4 V_{pp}, 100 Hz and 20 V_{pp}, 100 Hz (where the scale appears densest) as

an example. We used image processing software (ImageJ) to quantify the percentage area coverage (%) of $CaCO_3$, calculated by:

$$\% \text{ Area} = \frac{S_{\text{crystal}}}{S_{\text{image}}} \tag{2}$$

where S_{crystal} is the area of $CaCO_3$ crystals and S_{image} is the area of the SEM micrograph. The average $CaCO_3$ % area was nearly identical under these conditions (7.06±0.76% for the 4 V_{pp}, 100 Hz and 6.93±1.31% for 20 V_{pp}, 100 Hz (Fig. S4a). Also, the respective SEM micrographs showed very similar crystal structure and distribution (Fig. S4b).

In addition, surface contact angle measurements were used to quantify $CaCO_3$ scaling under different electrical conditions (Fig. S4c). The contact angle of the bare titanium surface (c1 in Fig. S4c) was measured to be 87.3±0.9°, which is consistent with previously reported values[7]. Calcite, with a lower contact angle (48° to 60°)[8], leads to a reduction in the contact angle due to $CaCO_3$ buildup, when present. The measured contact angles (c2-c9 in Fig. S4c) correspond well with the observed $CaCO_3$ coverage (Fig. 3d). Specifically, a higher contact angle of 75.2±2.7° was observed at 20 V_{pp}, 100 Hz, compared to 71.4±1.3° at 4 V_{pp}, 100 Hz, suggesting reduced $CaCO_3$ coverage at higher voltages. Also, nearly identical contact angles of 82.1±4.1°, 80.7±3.5°, 83.6±2.0° at 4 V_{pp} for 0.1,1, and 10 Hz, respectively, suggest minimal $CaCO_3$ coverage at these conditions. The low contact angles of 67.1±1.8° and 71.4±1.3° at 2 V and 4 V_{pp}, 100 Hz, respectively, are comparable to the 65.2±2.2° at 0 V, indicating a high $CaCO_3$ coverage similar to that seen in the absence of an applied potential.

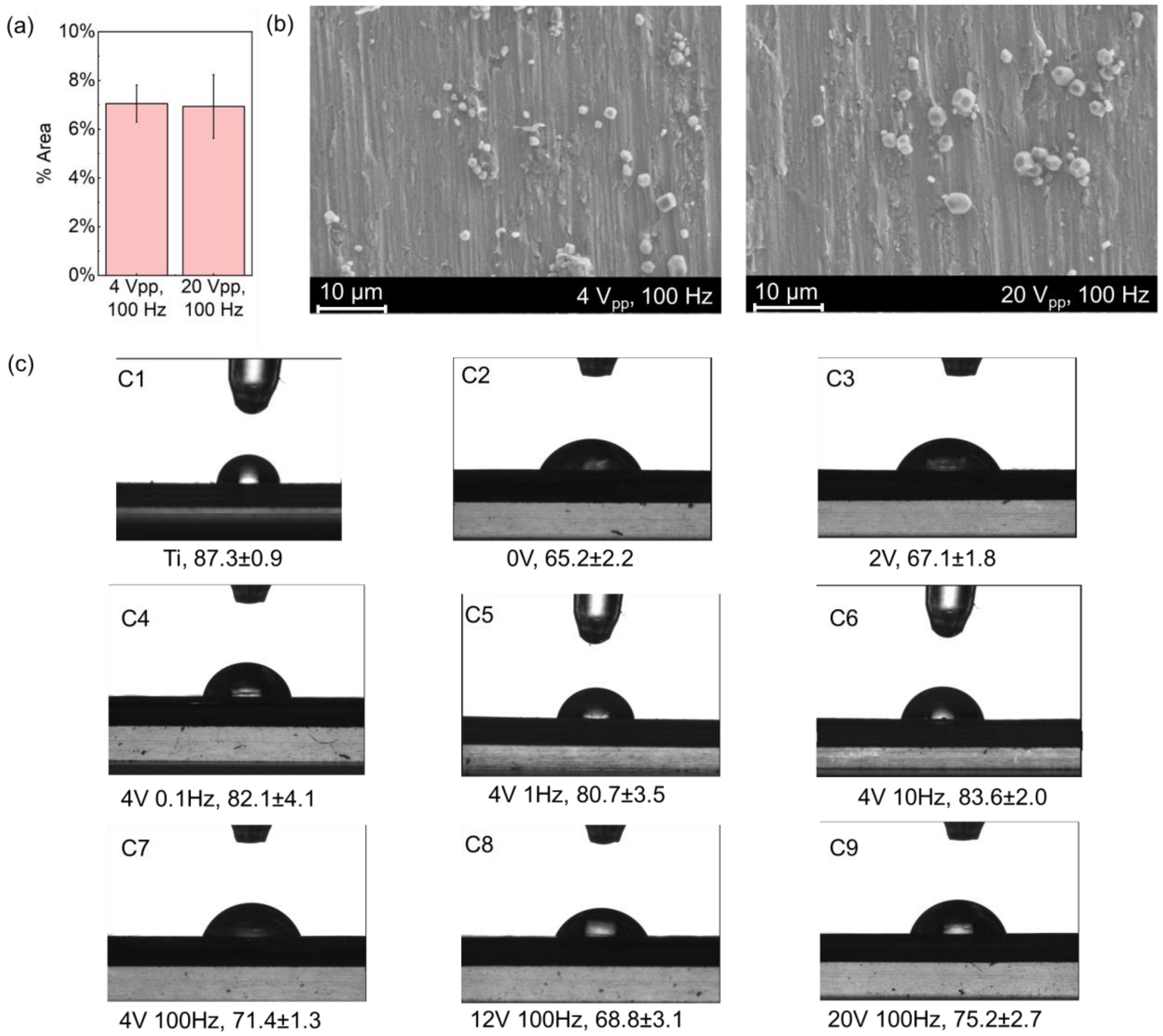

Supplementary Fig. 4. Characterization of individual electrode surface. **a**,**b**,**c**, Area % of $CaCO_3$ under 4 V_{pp}, 100 Hz and 20 V_{pp}, 100 Hz conditions (**a**), and SEM micrograph at 4 V_{pp}, 100 Hz and 20 V_{pp}, 100 Hz, at the same magnification (5000x) as that of Fig. 3e in the manuscript (**b**). The contact angles of the electrodes (c1-c9) under different electrical conditions (**c**).

The EDS mapping and spectrum of Ti, Ca, C, and O elements on the surface were analyzed under the conditions of 0 V and 4 V_{pp}, 1 Hz potential (Fig. S5a,b). Under the open circuit (0 V) conditions, the presence of Ca, C, and O in all the cubic-structured crystals confirmed that the composition of crystals was $CaCO_3$ and therefore the crystals are calcite (Fig. S5a). The elemental spectrum further suggested that the $CaCO_3$ on the electrode surface was qualitatively less than that of Ti (Fig. S5a), implying that the presence of $CaCO_3$ was not significant enough to hinder the effective area available for electron transfer. In comparison, under the 4 V_{pp}, 1 Hz AC conditions, the $CaCO_3$ crystals were much smaller in size (Fig. S5b). Furthermore, the

elemental analysis indicated a qualitatively reduced presence of $CaCO_3$ on the surface (Fig. S5b). These results highlighted the impact of applied AC potential on both the size and abundance of $CaCO_3$ crystalline formations.

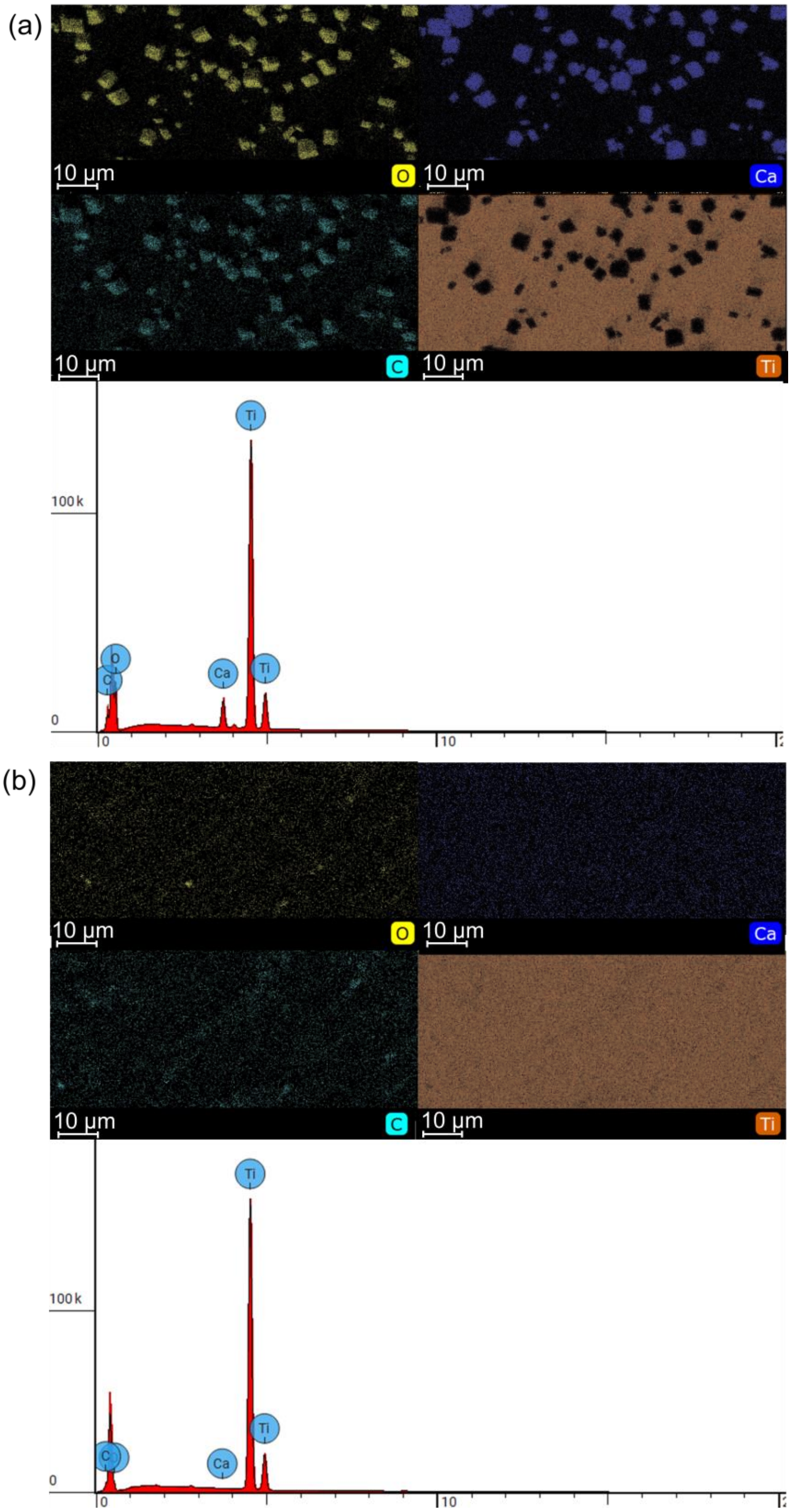

Supplementary Fig. 5. Elemental distribution on the electrode surface. a,b, EDS elemental mapping and spectrum of electrode surface under the conditions of no potential (**a**), and 4 V_{pp}, 1 Hz AC potential (**b**).

Supplementary Note 7. Comparison between measured and simulated electric currents

Based on the equivalent circuit of our system (Fig. 4a), the current under the experimental AC conditions was predicted. We took the average of all the two-hour datapoints and compared the predicted currents to measured currents. This comparison revealed a good match between predicted and measured currents (Fig. S6). At 4 V_{pp} from 1 to 100 Hz, our model predicts an increase in current from 7.4 to 30.4 mA. The actual measurements showed a similar trend, with currents increasing from 2.1±0.2 to 22.0±0.7 mA. When we increased the voltage from 4 to 20 V_{pp} at 100 Hz, the simulated currents increased from 30.4 to 147.9 mA, while the measured currents also increased from 22.0±0.7 to 109.4±1.6 mA. These suggest our model, while mostly accurate, tends to slightly overestimate the currents, possibly due to not fully accounting for the resistance at the interface and in the solution.

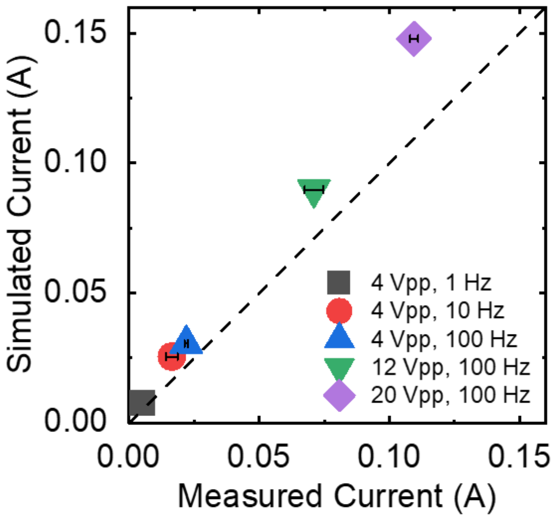

Supplementary Fig. 6. Simulated current as a function of measured current under the AC conditions.

Supplementary Note 8. Derivation of simplified Nernst-Planck equation

The simplification of Nernst-Planck equation for our experiment is based on the following assumptions. First, given that the solution was stirred at a slow speed of 250 rpm to achieve complete mixing only, the convection term of this equation was considered negligible and approximated to zero. Furthermore, the applied AC potential, as opposed to a DC potential of

the same voltage, primarily minimized the charge transfer through electrochemical reactions[9]. This condition significantly reduced the transport of charged species due to their consumption at the electrode, allowing us to also approximate the diffusion component to zero. After these simplifications, the Nernst-Plank equation can be represented as:

$$J = \frac{zFD}{RT} cE \tag{3}$$

Based on the charge balance across the external circuit and the electrolytic solution, we converted the electric current to the molar flux of charges and equated the charges carried by current to the charges carried by ions in molar flux, which is given by:

$$\frac{I}{AF} = J \tag{4}$$

where I (A) is the current and A (m^2) is the electrode area. Also, the Stokes-Einstein relations is applied to simplify the migration, which is determined by[10]:

$$D = \frac{\mu k_B T}{q} \tag{5}$$

where k_B (J K^{-1}) is the Boltzmann constant, T (K) is temperature, and q (C) is the electric charge of particle. By substituting Eqs. 6, S4, S5 into Eq. S3, the final form of simplified Nernst-Planck equation is expressed as:

$$\frac{I}{AF} = vzc \tag{6}$$

Supplementary Note 9. Brownian motion calculations

Brownian motion arises from the thermal fluctuations of particles themselves, which is distinct from motions caused by an external force. The Brownian movement is random because of constant collisions among the particles. To simplify the analysis of these complex interactions, historically two statistical mechanics models have been developed by Einstein[11] and Smoluchoski[12] to describe the Brownian motion. Remarkably, both models converge on

the same equation relating a particle's displacement to its diffusivity (Eq. 17). An alternative form of Eq. 17 can be written as:

$$t = \frac{2\overline{D}}{\overline{v_B}} \tag{7}$$

Based on this equation, we determine that the time scale when the observed Brownian velocity matches a typical displacement velocity of 7.32×10^{-6} m s^{-1} is 35 s. Only the collisions of nano-sized ions and ion clusters beyond a time scale of 35 s are disrupted. Within this time frame, the collisions of ions are not disrupted, but they did not form critical nuclei because this time is much less than the induction time (3600 s) established by the experiments. Therefore, the overall collision frequency is reduced, which leads to mitigation of nucleation.

Supplementary Note 10. Impact of EDL charging on currents

The EDL charging time is a function of ion concentrations and applied surface potential according to Eq. 14 of the main manuscript. In Fig. S7a, we specifically explored how changes in AC voltage affect the EDL charging time. By increasing the AC voltage from 4 to 20 V_{pp}, we observed a decrease in the EDL charging time from 0.121 to 0.049 s.

We further studied the importance of faster EDL charging time over other factor when analyzing the increase in current with voltage. The two factors are resistance and EDL charging time. The current as a function of decreasing charging time were studied not considering and considering the factor of resistance from 4 to 20 V_{pp} (Fig. S7b). It is clear that currents resulting from both the decrease in EDL charging time and the increase in voltage (accounting for resistance) are substantially higher than currents affected by charging time alone. Furthermore, the gap between two sets of currents widens with the decrease in charging time. Therefore, these results confirm that the increase in current with voltage is primarily due to the Ohm's law - the relationship between voltage, current, and resistance - instead of merely an expedited EDL charging.

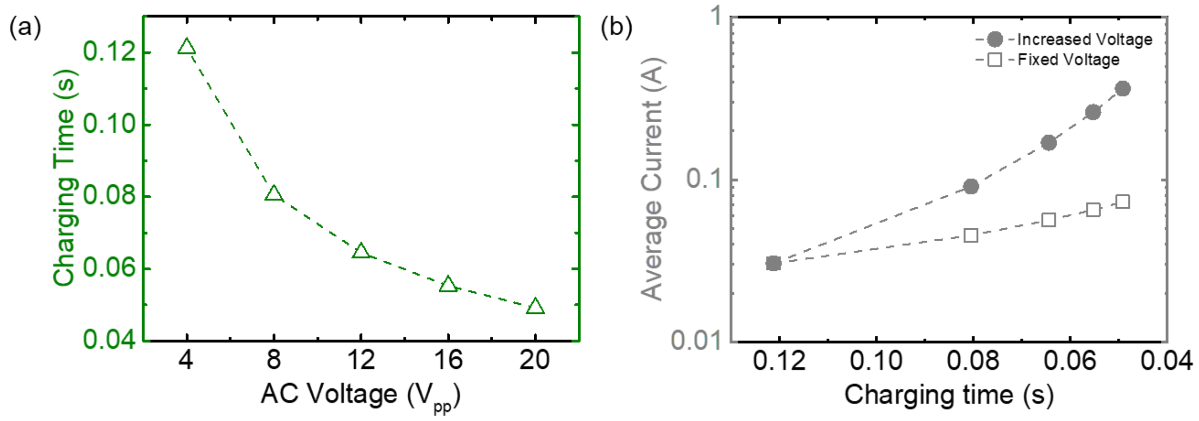

Supplementary Fig. 7. EDL charging behavior influenced by applied voltage. a, Charging time as a function of AC voltage. **b**, Average current as a function of decreasing charging time with fixed voltage of 4 V_{pp}, and increased voltages from 4 to 20 V_{pp}.

Supplementary Note 11. Physics-based crystallization model

The connection between A and turbidity of $CaCO_3$ can be effectively analyzed through a two-stage crystallization model inspired by an existing model for $BaSO_4$ crystallization[13]. Specifically, this model employs the same classical nucleation theory (CNT)[14], surface growth reaction[15,16], Smoluchowski aggregation theory[17], and Mie light theory[18] as the previous model to predict the time-dependent evolution of bulk solution turbidity, using the parameters of $CaCO_3$ crystallization. We simplify this model into an equation to show relationship between the turbidity and other independent variables, instead of reproducing the original, complex expression. The formula for time-dependent turbidity is:

$$\text{Turbidity}\ (t) = f(n_0, t_n, r_1(t)) \tag{8}$$

where n_0 (m^{-3}) is the number of critical nuclei per unit volume, t_n (s) is the induction time, and $r_1(t)$ (m) is the radius of post-critical nuclei, which is implicitly related to n_0, t_n, and surface growth rate (k_r, s). In addition, the expression for the nucleation kinetics by CNT provides an explicit relationship between n_0 and t_n, which is:

$$\ln(\frac{n_0}{t_n}) = \ln(A) - \frac{k_J}{(\log \mathrm{SI})^2} \tag{9}$$

where A (m^{-3} s^{-1}) is the pre-exponential factor known as collision frequency of Eq. 1, the slope of k_J is a dimensionless function of effective interfacial energy (γ, J m^{-2}) and temperature (T, K), and SI is dimensionless saturation index. Overall, the calculation of turbidity relies on two independent variables associated with nucleation and crystal growth, the induction time (t_n) and surface growth rate (k_r). In our model, all the values for the parameters are either extracted from the results of a previous $CaCO_3$ crystallization study[19], or determined based on our experimental conditions (Table S3). The values for t_n and k_r are obtained by fitting this model to the experimental measured turbidity values under the 0 V condition. Specifically, the unit of turbidity is converted from NTU into m^{-1} using a 1:1 ratio[20,21].

Supplementary Table 3. Values for different variables and parameters of the $CaCO_3$ crystallization model.

Name	Symbol (Unit)	Value	Source
Surface growth rate	k_r (m s^{-1})	8.30×10^{-6}	Fitted
Time of nucleation	t_n (s)	132	Fitted
Collision frequency	A (m^{-3} s^{-1})	6.34×10^{30}	Previous $CaCO_3$ Study
Slope	k_J	-53.19	Previous $CaCO_3$ Study
Logarithmic Saturation index	$\log \mathrm{SI}$	1.04	Experimental Condition

The A values are back calculated from simulated turbidity with fit of least square errors to experimental turbidity under different AC conditions (Table S4). Also, the relative turbidity values are listed to compare to the A values.

Supplementary Table 4. The turbidity and A values under different AC conditions.

Electrical Condition	A value (m^{-3} s^{-1})	Relative Turbidity
No potential	6.34×10^{30}	1
4 V_{pp}, 0.1 Hz	1.90×10^{29}	0.030
4 V_{pp}, 1 Hz	1.58×10^{29}	0.024
4 V_{pp}, 10 Hz	1.84×10^{29}	0.030
4 V_{pp}, 100 Hz	4.31×10^{30}	0.73
12 V_{pp}, 100 Hz	8.37×10^{30}	1.08
20 V_{pp}, 100 Hz	1.08×10^{29}	0.18

Supplementary References

1. Rao, U. *et al.* Mineral Scale Prevention on Electrically Conducting Membrane Distillation Membranes Using Induced Electrophoretic Mixing. *Environmental Science & Technology* **54**, 3678-3690, doi:10.1021/acs.est.9b07806 (2020).
2. Jung, B. *et al.* Impact of polarity reversal on inorganic scaling on carbon nanotube-based electrically-conducting nanofiltration membranes. *Chemical Engineering Journal* **452**, 139216 (2023).
3. Kim, J., Tijing, L., Shon, H. K. & Hong, S. Electrically conductive membrane distillation via an alternating current operation for zero liquid discharge. *Water Research*, 120510 (2023).
4. Dong, Q. *et al.* Electrospun composites made of reduced graphene oxide and activated carbon nanofibers for capacitive deionization. *Electrochimica Acta* **137**, 388-394 (2014).
5. Laxman, K., Myint, M. T. Z., Bourdoucen, H. & Dutta, J. Enhancement in ion adsorption rate and desalination efficiency in a capacitive deionization cell through improved electric field distribution using electrodes composed of activated carbon cloth coated with zinc oxide nanorods. *ACS applied materials & interfaces* **6**, 10113-10120 (2014).
6. Li, L., Zou, L., Song, H. & Morris, G. Ordered mesoporous carbons synthesized by a modified sol–gel process for electrosorptive removal of sodium chloride. *Carbon* **47**, 775-781 (2009).
7. Lim, Y. J., Oshida, Y., Andres, C. J. & Barco, M. T. Surface characterizations of variously treated titanium materials. *International Journal of Oral & Maxillofacial Implants* **16** (2001).
8. Bikkina, P. K. Contact angle measurements of CO2–water–quartz/calcite systems in the perspective of carbon sequestration. *International Journal of Greenhouse Gas Control* **5**, 1259-1271 (2011).
9. Bard, A. J., Faulkner, L. R. & White, H. S. *Electrochemical methods: fundamentals and applications*. (John Wiley & Sons, 2022).
10. Rabiller-Baudry, M., Chaufer, B., Aimar, P., Bariou, B. & Lucas, D. Application of a convection–diffusion–electrophoretic migration model to ultrafiltration of lysozyme at different pH values and ionic strengths. *Journal of membrane science* **179**, 163-174 (2000).
11. Einstein, A. Über die von der molekularkinetischen Theorie der Wärme geforderte Bewegung von in ruhenden Flüssigkeiten suspendierten Teilchen. *Annalen der physik* **4** (1905).
12. Smoluchowski, M. Sur le chemin moyen parcouru par les molécules d'un gaz et sur son rapport avec la théorie de la diffusion. *Pisma Mariana Smoluchowskiego* **1**, 479-489 (1924).
13. Dai, Z. *et al.* Two-stage model reveals barite crystallization kinetics from solution turbidity. *Industrial & Engineering Chemistry Research* **58**, 10864-10874 (2019).
14. Nielsen, A. E. Kinetics of precipitation. *(No Title)* (1964).
15. Mullin, J. W. *Crystallization*. (Elsevier, 2001).
16. Davis, T. M. *et al.* Mechanistic principles of nanoparticle evolution to zeolite crystals. *Nature materials* **5**, 400-408 (2006).
17. Smoluchowski, M. Drei Vorträge über Diffusion, Brownsche Molekularbewegung und Koagulation von Kolloidteilchen (Three Lectures on Diffusion, Brownian Motion, and Coagulation of Colloidal Particles), Phys. Z., 17, 557 (1916); Versuch einer Mathematischen Theorie der Koaguationskinetik Kolloider Lösugen (Trial of a

Mathematical Theory of the Coagulation Kinetics of Colloidal Solutions). *Z. Physik. Chem* **92**, 129-155 (1917).

18 Berg, J. C. *An introduction to interfaces & colloids: the bridge to nanoscience*. (World Scientific, 2010).

19 Koutsoukos, P. G. & Kontoyannis, C. G. Precipitation of calcium carbonate in aqueous solutions. *Journal of the Chemical Society, Faraday Transactions 1: Physical Chemistry in Condensed Phases* **80**, 1181-1192 (1984).

20 Kirk, J. Estimation of the scattering coefficient of natural waters using underwater irradiance measurements. *Marine and Freshwater Research* **32**, 533-539 (1981).

21 Peng, F., Effler, S. W., Pierson, D. C. & Smith, D. G. Light-scattering features of turbidity-causing particles in interconnected reservoir basins and a connecting stream. *water research* **43**, 2280-2292 (2009).
